# Multiple Pregnancy: Epidemiology and Association with Maternal and Perinatal Morbidity

**DOI:** 10.1055/s-0038-1668117

**Published:** 2018-09

**Authors:** Danielly Scaranello Santana, Fernanda Garanhani Surita, José Guilherme Cecatti

**Affiliations:** 1 Department of Ginecology and Obstetrics, Faculty of Medical Sciences, Universidade de Campinas, Campinas, SP, Brazil

**Keywords:** multiple pregnancies, maternal morbidity, perinatal morbidity, high-risk pregnancy, gestações múltiplas, morbidade materna, morbidade perinatal, gestação de alto risco

## Abstract

Twin pregnancy accounts for 2 to 4% of total births, with a prevalence ranging from 0.9 to 2.4% in Brazil. It is associated with worse maternal and perinatal outcomes. Many conditions, such as severe maternal morbidity (SMM) (potentially life-threatening conditions and maternal near-miss) and neonatal near-miss (NNM) still have not been properly investigated in the literature. The difficulty in determining the conditions associated with twin pregnancy probably lies in its relatively low occurrence and the need for larger population studies. The use of the whole population and of databases from large multicenter studies, therefore, may provide unprecedented results. Since it is a rare condition, it is more easily evaluated using vital statistics from birth *e-*registries. Therefore, we have performed a literature review to identify the characteristics of twin pregnancy in Brazil and worldwide. Twin pregnancy has consistently been associated with SMM, maternal near-miss (MNM) and perinatal morbidity, with still worse results for the second twin, possibly due to some characteristics of the delivery, including safety and availability of appropriate obstetric care to women at a high risk of perinatal complications.

## Resumo

A gestação gemelar é responsável por 2 a 4% do total de nascimentos, com uma prevalência variando de 0,9 a 2,4% no Brasil. Ela é associada a piores resultados maternos e perinatais. Muitas condições, como a morbidade materna grave (condições potencialmente ameaçadoras da vida e *near-miss* materno) e *near-miss* neonatal ainda não foram investigadas de forma apropriada na literatura. A dificuldade na determinação de condições associadas com a gestação gemelar provavelmente reside em sua ocorrência relativamente baixa e na necessidade de estudos populacionais maiores. O uso da população total e de bancos de dados de grandes estudos multicêntricos podem então fornecer resultados sem precedentes. Considerando que esta é uma condição rara, ela é mais facilmente avaliada usando estatísticas vitais de registros eletrônicos de nascimento. Portanto, realizamos uma revisão da literatura para identificar as características da gestação gemelar no Brasil e no mundo. A gestação gemelar está consistentemente associada à morbidade materna grave, ao *near-miss* materno e à morbidade perinatal, com resultados ainda piores para o segundo gemelar, possivelmente devido a algumas características da atenção ao nascimento, incluindo segurança e disponibilidade de cuidados obstétricos apropriados para a mulher com alto risco de complicações perinatais.

## Introduction

Twin pregnancies account for 2 to 4% of the total number of births.^1^ ^2^ ^3^ ^4^ ^5^ ^6^ Spontaneous twin pregnancy rates vary worldwide. The prevalence rates range from less than 8 twin pregnancies per 1,000 births in the East, Southeast and Southern Asia, India, and Oceania, 9–16 per 1,000 births in the United States and Latin America, to 17 or more per 1,000 births in Africa.^7^ The highest rates of twin pregnancies are found in Nigeria and the lowest rates occur in Japan.^8^ This difference is mainly due to dizygotic twin pregnancies, since the prevalence of monozygotic pregnancies is practically constant, ranging from 3.5 to 4 per 1,000 births.^7^ ^8^

Twin pregnancy rates have increased in the past 30 years, particularly in high-income or middle-income countries, owing to a more advanced maternal age to become pregnant, a decline in fertility and an increased use of assisted reproductive techniques.^2^ ^3^ ^4^ It is well-known that twin pregnancy is associated with higher maternal and perinatal risks. The maternal adaptation to a twin pregnancy leads to several complications. Maternal death (MD) associated with a twin pregnancy is 2.5-fold higher than in a singleton pregnancy.^4^ The rate of perinatal mortality is two to three times higher in twins than among singleton newborn infants, primarily due to preterm birth, fetal growth restriction (FGR), low birth weight (LBW) and intrapartum anoxia.^9^ ^10^

Maternal morbidity and mortality associated with twin pregnancy have not been appropriately discussed in the literature, since there are few studies on the topic. The few existing studies have methodological limitations, and a small number of cases.^1^ ^2^ ^3^ ^11^ Studies on mortality and morbidity are rare, but even rarer are studies that associate twin pregnancy with the new concepts of severe maternal outcome (SMO) and maternal near-miss (MNM). The objective of the present study is to introduce aspects associated with the epidemiology of twin pregnancy, highlighting not only the clinical aspects, already very well described in the literature, but also maternal and neonatal morbidity and near-miss issues that are much less studied.

## Etiology and Epidemiology of Twin Pregnancy

Twin pregnancy may result from the fertilization of two oocytes by two sperms, generating dizygotic twins, or from the fertilization of a single oocyte that will subsequently split into two similar structures, each capable of developing an individual, generating monozygotic twins.^6^ ^12^

Dizygotic pregnancies are the majority and occur spontaneously due to an increased concentration of follicle-stimulating hormone (FSH) in the woman.^6^ Therefore, the risk factors for its occurrence are: geography (it occurs more frequently in countries with milder climate),^6^ ethnicity (black ethnicity),^8^ multiparity,^6^ ^8^ advanced maternal age (ovarian hyperstimulation due to increased gonadotrophins between the ages of 35 and 39 years old),^5^ ^6^ ^8^ low socioeconomic condition,^8^ use of oral contraceptives,^8^ family history (7–15% of the population have a dominant gene for twin pregnancy),^6^ ^8^ and use of assisted reproductive techniques.^5^ ^6^ ^8^

Monozygotic pregnancies occur in 30% of twin pregnancies and are widely determined by genetic factors. In vitro fertilization is a risk factor for monozygotic pregnancies, since the embryo procedures may generate an alteration in the zona pellucida.^8^ ^12^ ^13^ ^14^ Contrary to dizygotic pregnancies, which are always dichorionic, the chorionicity in monozygotic pregnancies is determined by the time of the division of both cell masses. Should the division occur in the first 72 hours after the fertilization, the pregnancy is dichorionic and diamniotic. Should the division occur between days 4 and 8, the pregnancy is monochorionic and diamniotic. Should the division occur after the eighth day, the pregnancy is monochorionic and monoamniotic.^6^ ^12^ About 75% of the monozygotic pregnancies are monochorionic and, among the monochorionic pregnancies, ∼ 2% are monoamniotic.^13^ ^15^

The chorionicity is evaluated by an ultrasonography performed early in the pregnancy, within the first 13 weeks of gestation. The lambda sign, typical of dichorionic pregnancies, is detected.^12^ ^16^ It is important to identify the chorionicity, owing to the occurrence of complications that are most commonly associated with monochorionic pregnancies: abortion (3 times more frequent); congenital malformations and chromosomal disorders, which occur in 2% of the twin pregnancies; minor malformations, which have an incidence of 4%; weight discordance; preterm birth and LBW, consequently with increased perinatal mortality and morbidity, which are 3 to 10 times higher in monochorionic pregnancies due to the chorionicity.^13^ ^14^ ^15^ ^16^

Monochorionic pregnancies are associated with specific conditions. The incidence of twin-to-twin transfusion syndrome (TTTS) is 10 to 20% in monochorionic pregnancies.^17^ In TTTS, a communicating unidirectional flow occurs between the fetuses, through deep arteriovenous anastomoses and superficial venovenous and arterioarterial anastomoses, with repercussions for both fetuses. It is clinically manifested by a donor twin with severe growth restriction, anemia, and oligohydramnios, and a recipient twin affected by circulatory overload with polycythemia, cardiac complications, hydrops, and polyhydramnios. Selective FGR occurs in 10 to15% of all monochorionic pregnancies and is diagnosed by a difference of weight higher than 25% between the fetuses and one fetus with weight below the tenth percentile, associated with an increased perinatal morbidity and mortality.^18^ When one twin dies, the risk of death or neurologic sequelae for the other fetus is very high due to vascular anastomoses, requiring periodic ultrasonographic monitoring. Umbilical cord accidents are a specific condition of monoamniotic pregnancies that occur in 48 to 80% of the cases and are associated with high rates of perinatal mortality.^12^ ^13^ ^15^

## Twin Pregnancy in Brazil

Few studies have adequately assessed twin pregnancies in Brazil. The existing studies have investigated specific locations, and twin pregnancy was not characterized by regions. The oldest prevalence data (1984–1996) was identified in a study with a small population (116,699 deliveries) assessing perinatal mortality in comparison to singleton pregnancies. In this study, a survey in the largest maternity hospital in Campinas, Saõ Paulo state, Brazil, identified a prevalence of 0.9% twin births.^19^ Another small study reported 7,997 deliveries in a private hospital in São Paulo, São Paulo state, Brazil, from 1995–1998, identifying a prevalence of 24.02 twin deliveries per 1,000 births, of which 19.51‰ were dizygotic pregnancies and 2.13‰ were triplet pregnancies. In this study, there was an increase in the prevalence of dizygotic pregnancies (13.51 in 1995–28.98‰ in 1998), possibly due to the advanced maternal age, multiparity and in vitro fertilization.^20^

Using the Brazilian Information System on Live Births (SINASC, in the Portuguese acronym) database, two studies were published including populations from different states. The first study investigated multiple births in Porto Alegre, Rio Grande do Sul state, Brazil, from 1994 to 2005, in a population of 263,252 births, and the prevalence of multiple pregnancies was 2.1%. In the periods studied, the rate increased 24.7% for twin pregnancies and 150% for triplet pregnancies or pregnancies with more fetuses. Twin pregnancies were more frequent in women with higher levels of school education, advanced age and deliveries in private hospitals, possibly suggesting a higher use of assisted reproductive techniques.^21^ The second study investigated births in the city of São Paulo, São Paulo state, Brazil, from 2003 to 2014, identifying 24,589 (11.96–7.5‰ dizygotic and 4.42‰ monozygotic) twin births and 736 (0.36‰) triplet or more fetuses in a total of 2,056,016 births. Older maternal age was a factor strongly associated with twin pregnancies, particularly dizygotic pregnancies, as well as other factors such as body mass index (BMI) and air pollution.^22^

In Brazil, there is an interesting fact about a place called “Twin City.” Cândido Godói is a small city of ∼ 6,000 inhabitants in the Rio Grande do Sul state, with a high rate of twin births (2% from 1994–2006). In Linha de São Pedro, a subdistrict of the city, the twin birth rates reached 10% in 1994, generating widespread assumptions. One was a folkloric belief that Nazi studies may have been conducted in this population by Joseph Mengele. Two different studies evaluated this population to find the reasons for the high prevalence of twinning. Twin pregnancies were strongly associated with genetic conditions in that population. Most specifically, genetic polymorphisms in the p53 pathway, responsible for blastocyst implantation and maintenance of the embryo within the uterus, played a role.^23^ ^24^

## The Importance of Vital Records in Rare Conditions such as Twin Pregnancies

Twin pregnancy is a rare condition that should be considered in vital statistics assessments. Vital statistics refer to continuous routine birth and death registries in a certain population. These registries can be integrated into a national surveillance program, in which rare conditions can be identified. Rare conditions are hardly identified in sample analyses but are easily identified in national-scale analyses.

Health records allow the surveillance and investigation of mortality, contributing to population-based indicators, such as fertility and mortality, by assessing the participation of individuals in economic, social, political life, safety and sustainability. From the birth registries, people are recognized and counted, broadening government responsibility and maximizing the access to human rights for the most vulnerable and marginalized population. Registries provide a basis for decision-making in public health policies that also involve social issues and enable the development of interventions with better financial management and universal health care coverage.^25^ ^26^ ^27^

Despite its importance, this type of registry remains neglected.^27^ It is estimated that 1 in every 3 children aged ≤ 5 years worldwide does not have a birth record, and two-thirds of deaths were not registered or counted in vital records. More than half of the World Health Organization (WHO) member states have no mortality data or their data are of inferior quality, with little value for public health policies or planning.^28^ These countries use indirect techniques to identify these events or use a sampling method in research. However, the sample is not always sufficient to determine rare events, and the indicators may not be interpreted as population-based parameters because there may be limitations in the sampling design.^26^ ^29^

Data obtained in vital statistics and population-based databases enable the creation of the so-called *e*-registries (electronic registries), information systems and storage technologies, as well as the analysis and dissemination of health data. These systems have assumed importance because the global health agencies are supporting more sustainable and safer ways to obtain and disseminate health information The aim of the *e*-registries is to unify information from the preconception to the postpartum period and newborn and child health data. This population-based collection has less information bias and its data validity is higher. These registries are an emergent opportunity for researchers in maternal health, although middle- and low-income countries still have an insufficient data collection, analysis, and notification of health data, resulting in incomplete and fragmented data.^30^ ^31^

Many countries have databases containing birth records. Norway has the Medical Birth Registry of Norway (MBRN). The United States has the National Center for Health Statistics (NCHS), and Brazil has the Health Informatics Department of the Brazilian Ministry of Health (DATASUS, in the Portuguese acronym), which stores the SINASC data. The SINASC is a birth registry of the entire Brazilian population that has been gradually implemented since 1994. Its aim is to gather epidemiological birth data that is informed throughout the national territory and provide birth data for all levels of the health care system.^32^

### Perinatal Outcomes in Twin Pregnancy

There are several perinatal complications associated with a twin pregnancy, although the worst outcome is perinatal death. Perinatal death is defined as the sum of fetal deaths (intrauterine death of any product after 22 completed weeks or 500 g in weight) and deaths of live births in the first 7 days after birth. Twin pregnancies, when compared to singleton pregnancies, increase two to three times the risk of perinatal death. Preterm delivery and LBW are the most important factors for determining these perinatal outcomes.^7^ ^9^ ^33^ ^34^

Preterm birth has a prevalence ranging from 5 to 18% in different countries. Brazil, India, China, Nigeria and the United States are among the 10 countries with the highest estimated number of preterm births.^35^ ^36^ ^37^ ^38^ ^39^ Preterm births occurred in 51% of the twin pregnancies and early preterm births (birth at < 32 weeks) occurred in 14% of the twin pregnancies.^4^ ^10^ ^40^

Preterm birth is directly associated with an increased risk of neonatal death and morbidity. Major causes of preterm birth are preterm delivery, premature rupture of membranes, maternal conditions (hypertension, diabetes, placental abruption) and fetal conditions that lead to preterm delivery (FGR, fetal distress, the death of one twin). Morbidity associated with preterm birth refers mainly to respiratory distress, intraventricular hemorrhage and necrotizing enterocolitis.^34^ ^41^ ^42^ Neonatal morbidity seems to be more important when there is weight discordance between both fetuses, with a higher likelihood of intracranial hemorrhage and patent ductus arteriosus.^12^

Low birth weight, defined as weight < 2,500 g at birth, occurs in half of the cases of twin pregnancy, due to preterm delivery and FGR.^12^ Among the causes of growth restriction and weight discordance are unequal placentation and uterine overload, with different blood flow and nutrients for the fetuses, genetic differences, relative placental insufficiency, cord insertion abnormalities, malformations, and infection. Twin and singleton pregnancies appear to be similar in growth until ∼ 30 weeks, when the twins are smaller than fetuses from singleton pregnancies. Between 34 and 35 weeks, the difference in fetal weight is clear and the incidence of FGR at 38 weeks quadruples, including virtually half of the twin births.^4^ ^10^ ^12^ ^40^ Nevertheless, growth evaluation is usually based on growth curves established by singleton pregnancies. Several studies have recommended the creation of growth curves specific to twins or the use of some already existent curves for twin infants.^43^ ^44^ ^45^ ^46^

Among the unfavorable outcomes are fetal death and neonatal death. The fetal death rate is higher among twin pregnancies than in singleton pregnancies. In 2009, it was estimated that this complication occurred in 12.3 per 1,000 twin births, while in single pregnancies it occurred in 5 per 1,000 births.^4^

Recently, the concept of neonatal near-miss (NNM) has also emerged. It is a new marker of severity that is similar to maternal near-miss (MNM) that enables the identification of a group of newborn infants at a higher risk of neonatal death. Neonatal near-miss is defined as a severe complication that almost resulted in the death of a newborn infant during the neonatal period (the first 28 days of life). As MNM, NNM has a higher incidence than neonatal death.^47^ ^48^ ^49^ Since it is a very new concept, the majority of articles published still discusses the diagnostic criteria for its identification. An article published in 2015 defined two sets of criteria for identifying NNM cases: pragmatic criteria and management criteria, shown in Table 1. Based on these criteria, a systematic review was conducted, identifying an NNM rate that ranged from 21.4 to 72.5 per 1,000 live births. No other study has evaluated the association between NNM and twin pregnancy until the present.^47^ ^48^ ^49^

**Table 1:** Diagnostic criteria for neonatal near miss

Neonatal near-miss: at least one of these criteria
Pragmatic diagnostic criteria
Birthweight < 1,750 g
Apgar score < 7 at the 5^th^ minute
Gestational age < 33 weeks
**Management criteria**
Use of intravenous antibiotics
Nasal CPAP
Any intubation in the first 7 days
Use of phototherapy in the first 24 hours
Cardiopulmonary resuscitation
Use of any vasoactive drug
Use of anticonvulsants
Use of surfactant
Transfusion of blood derivatives
Use of corticosteroid for treatment of refractory hypoglycemia
Any surgical procedure

Abbreviations: CPAP, continuous positive airway pressure,

Source: Modified from Santos et al. (2015).^47^ ^48^

### Maternal Morbidity Associated with Twin Pregnancy

Maternal morbidity is associated with the maternal adaptation to physiological alterations that occur during a twin pregnancy.^12^ ^40^ In the first trimester, due to the increased levels of gonadotrophic hormone (hCG), nausea and vomiting occur more frequently, as well as hyperemesis gravidarum. A greater expansion of blood volume also occurs (in 40–50% of the single pregnancies and in 50–60% of the twin pregnancies), with hemodilution anemia and cardiovascular alterations, further exacerbated when related to preeclampsia and pulmonary edema.^4^ ^9^ ^12^ ^40^

Twin pregnancies are associated with a 2-fold to 3.5-fold higher risk of hypertensive alterations (preeclampsia, eclampsia, hemolysis, elevated liver enzymes, low platelet count [HELLP] syndrome and fatty liver of pregnancy) than singleton pregnancies, which present an incidence of 12.9 to 37%, mainly after the 20^th^ week of gestation.^9^ ^12^ ^50^ ^51^ The higher production of Human Placental Lactogen (HPL) in twin pregnancies causes insulin intolerance. In association with other factors such as weight gain, maternal age, and BMI, this could lead to gestational diabetes.^9^

Regarding local alterations, uterine overdistension is observed, generating an organ compression that may lead to urologic obstructive disorders and urinary tract infection, in addition to preterm labor (PTL), placental abruption and premature rupture of membranes (PROM). Furthermore, postpartum complications such as uterine atony and postpartum hemorrhage may also occur.^12^ ^40^

Despite all the recommendations of vaginal birth for twin pregnancies, even under ideal conditions, when the first twin is in cephalic presentation and weighs more than 1,500 g, 75 to 80% of these pregnancies still result in cesarean deliveries. The literature shows evidence that cesarean deliveries do not reduce complications such as neonatal sepsis, fetal distress for the second twin, or preterm delivery. In contrast, they increase the risk of postpartum hemorrhage, hysterectomy, blood transfusion, and complications due to placenta previa, placental accreta and placental abruption.^2^ ^3^ ^9^ ^52^ ^53^ ^54^ ^55^

Maternal mortality (MM) is the most severe complication associated with a twin pregnancy. The literature reports a 2.5 times higher incidence of MM is in twin pregnancies than in single pregnancies.^4^ ^34^ Maternal morbidity is very important in twin pregnancy. However, even more important is severe maternal morbidity (SMM), a marker of obstetric care that precedes and shares many characteristics with maternal death (MD). It is defined as the sum of cases of maternal near-miss and potentially life-threatening conditions (PLTCs).^56^ Maternal near-miss is defined by the WHO as a woman who almost died but survived complications during pregnancy, childbirth, or within 42 days of the termination of the pregnancy.^56^

**Figure 1:**
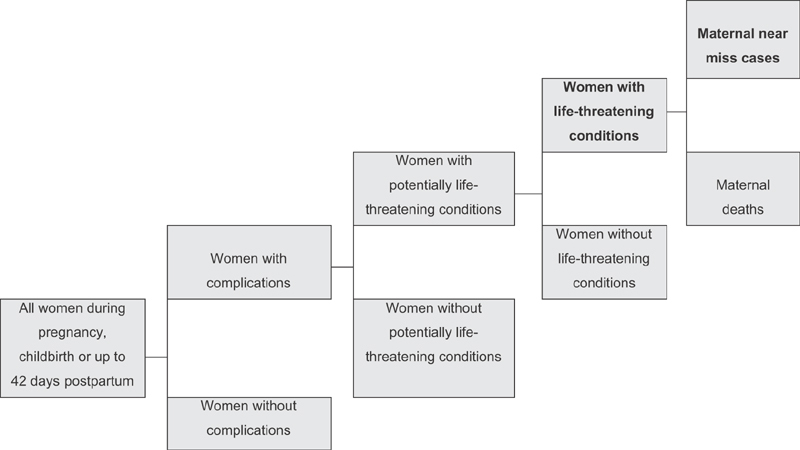
The continuum of maternal morbidity: from uncomplicated pregnancies to maternal death.

A chain of severe maternal events may culminate in the extreme event of MM. In this chain of events, the pregnancy may be complicated or not. Complicated pregnancies may threaten a woman's life and be a PLTC. In the latter, the woman may recover, have temporary or permanent incapacity, or die.^56^ Severe maternal morbidity represents the set of possible results for PLTCs (Figure 1).^57^

The diagnostic criteria for these conditions, shown in Table 2, were defined in 2009 by the WHO, who elaborated a list of PLTCs and 3 sets of criteria for MNM: clinical (capable of identifying severe cases essentially by using clinical judgment, without the need of special techniques or of specific laboratory exams), laboratory (specific laboratory alterations in diverse organs or system dysfunctions) and management criteria. Prior to this, the cases were identified by the so-called pragmatic criteria, which consisted of the presence of at least one of the following conditions: admission in an intensive care unit (ICU), blood transfusion, hysterectomy, and eclampsia.^57^ ^58^ ^59^ ^60^

**Table 2:** Definition criteria for severe maternal morbidity according to the World Health Organization.

Potentially life-threatening conditions			
Hemorrhagic disorders	Hypertensive disorders	Other systemic disorders	Severe management indicators
Abruptio placentae Placenta accreta/increta/percreta Ectopic pregnancy Postpartum hemorrhage Ruptured uterus	Severe preeclampsia Eclampsia Severe Hypertension Hypertensive encephalopathy HELLP syndrome	Endometritis Pulmonary edema Respiratory failure Seizures Sepsis Shock Thrombocytopenia < 100,000 Thyroid crisis	Blood transfusion Central venous access Hysterectomy ICU admission Prolonged hospital stay (7 days) No anesthetic intubation Return to operating room Surgical intervention
**Maternal near-miss: women who almost die, but survive a complication during pregnancy or childbirth within 42 days after birth**			
Clinical criteria	Laboratory-based criteria		Management based criteria
Acute cyanosis Gasping Respiratory rate > 40 or < 6/min Shock Oliguria non-responsive to fluids or diuretics Clotting failure Loss of consciousness lasting ≥12 hours Loss of consciousness and absence of pulse/heart beat Stroke Uncontrollable fit/total paralysis Jaundice in the presence of preeclampsia	Oxygen saturation < 90% for ≥60 minutes PaO_2_/FiO_2_ < 200 mm Hg Creatinine ≥ 300 µmol/l or ≥ 3.5 mg/dl Bilirubin > 100µmol/l or 6.0 mg/dl pH < 7.1 Lactate > 5 Acute thrombocytopenia (< 50,000 platelets) Loss of consciousness and the presence of glucose and ketoacidosis in urine		Use of continuous vasoactive drugs Hysterectomy following infection or hemorrhage Transfusion of ≥5 red cell units Intubation and ventilation for ≥60 minutes not related to anesthesia Dialysis for acute renal failure Cardio-pulmonary resuscitation
**Severe maternal outcome: refer to all cases of maternal near miss and maternal death**			
**Maternal death**: **death of a woman while pregnant or within 42 days of the termination of the pregnancy**			

Abbreviation: HELLP, hemolysis, elevated liver enzymes, low platelet count.

Source: Modified from Say et al. (2009).^56^

The WHO, in addition to determining the criteria for the identification of SMO cases, also proposed indicators to monitor the quality of obstetric care in MNM and MM cases. These indicators may be used to monitor the performance of care offered in health care units to women with complications.^56^ ^61^ ^62^

There has been an increasing interest in the subject, although until 2011 the prevalence of SMO was widely variable in the literature, mainly due to the use of non-standardized criteria for the identification of cases, with a rate of MNM ranging from 0.01 to 14.98%, depending on the clinical criterion used to identify SMO cases. The use of unique diagnostic criteria enables the identification and monitoring of MNM cases with the proposal of interventions required for its prevention.^57^ ^60^ ^61^ ^62^ ^63^ ^64^ ^65^ Thus, many recent studies that used the WHO criteria were capable of identifying the prevalence of MNM cases in a more uniform manner.^66^ ^67^ ^68^

Few studies have investigated the association between SMM and twin pregnancy, possibly because it still is a relatively new concept. However, the WHO Global Survey on Maternal and Perinatal Health (WHOGS), a cross-sectional multicenter study that evaluated more than 6,000 twin pregnancies and identified MNM by using pragmatic diagnostic criteria, concluded that twin pregnancy is a significant risk factor for maternal and perinatal morbidity when compared to single pregnancy in middle- or low-income countries.^34^ Until recently, the WHOGS was the largest and most complete assessment available of the relationship between twin pregnancy and SMO.

It is well known that twin pregnancy is associated with several maternal and fetal complications. Its incidence has increased in the last decades, making the condition an important object of study in the clinical practice. Nevertheless, it is difficult to obtain a database with a significant number of twin pregnancies. The use of large databases may provide surprising results regarding SMM, perinatal outcomes and NNM. As previously mentioned, few studies have evaluated SMM associated with twin pregnancy. Knowledge of this association may help us understand the severity of twin pregnancy for the woman, identify risk factors and enable the diagnosis of early signs of potentially life-threatening conditions. The investigation of NNM in twin pregnancies may be unprecedented, but the characterization of perinatal outcomes may modify the care approach in twin pregnancies.

### Delivery in Twin Pregnancy

In the past, there was much discussion about the best route of delivery in twin pregnancies, primarily for the second twin, who appeared to have the worst outcomes when the delivery route was vaginal.^53^ ^69^ However, multicenter studies currently provide strong evidence that vaginal delivery is safe when the first twin is in cephalic presentation.^70^ ^71^ Despite the evidence, cesarean section (CS) is the main delivery route in twin pregnancies, and the literature reports a prevalence ranging between 34 and 82%.^34^ ^53^ ^55^ ^72^ ^73^ As occurs in vaginal delivery, labor induction has also been shown to be safe, but its prevalence is still very low.^74^ It was observed that the prevalence of cesarean delivery in twin pregnancies is elevated, irrespective of the population-based sample evaluated, particularly in Brazil.

The WHO recommends that the CS rates do not exceed 10 to 15% of the total number of deliveries, since higher rates of cesarean deliveries are not associated with a reduction in maternal or neonatal mortality. In contrast, high CS rates may be associated with worse maternal results. These results raise doubts as to the safety of twin deliveries, diagnostic delays, and treatment of complications. Nevertheless, we should think about the possibility of inadequate care, considering scientific evidence-based management. Further studies are important to better understand the profile of twin pregnancy and its management. Twin pregnancy is a high-risk condition that requires adequate prenatal care to obtain the best possible maternal and perinatal outcomes.^4^ ^35^

### Particularities in Statistical Analysis of Twin Pregnancies

Little has been discussed about the statistical approach to twin pregnancy, and analyses are performed in a heterogeneous manner. Studies that use a mixed population of single and twin pregnancies often face difficulties in determining the sample. Efforts should be made to obtain a standardized analytical approach to be used in studies focusing on twin pregnancy.

Data collection instruments are often inadequate. The twin pregnancy is identified, but the data may be deficient or incomplete, especially for the second twin, whose data are frequently entered descriptively in an open field in the research clinical form. Chorionicity is easy to evaluate clinically. However, differently from assisted reproductive techniques, it may not be questioned in studies that interview women. Therefore, this information is not frequently assessed and would be of great importance, especially for perinatal outcomes.

The first difficulty in twin pregnancy lies in the rarity of the condition. Therefore, many studies generate results without statistical significance. The use of large databases and multicenter studies should be encouraged to assess rare conditions such as twin pregnancies. Databases such as the Brazilian SINASC exist and are often in the public domain. Data are available, but the information is being underused.

On a more specific statistical analysis, the identification of the study population may hinder the assessment of twin pregnancy. In a study where the woman/pregnancy is the focus, the number of live births from twin pregnancies is not always clear. In a study where the newborn infant is the focus, the number of women/pregnancies is rarely explicit. The number of live twin births is not always clear and does not simply correspond to twice the number of pregnancies, since triple births or those of a higher order may obviously occur. Furthermore, there may also occur fetal deaths. An estimate of the number of live births can be made, which is fundamental to calculate health indicators, and may specifically guide the estimation of twin pregnancies. This situation is yet to be better discussed in the literature.

Fetal weight is also evaluated in a customized manner on the analysis of similar studies, as previously mentioned. The use of specific curves for twin fetuses and newborn infants would be ideal. However, it is also possible to use curves that represent characteristics of the study population. Small-for-gestational-age (SGA) fetuses can be identified. Small-for-gestational-age is a condition that corresponds to the concept of fetal growth restriction. In addition, other curves may be used and should be considered, in an attempt to encompass the conditions associated with a twin pregnancy.

Another difficulty in the analysis of twin pregnancies concerns the assessment of newborn vitality, commonly expressed by a 5-minute appearance, pulse, grimace, activity, respiration (Apgar) score < 7. In multiple pregnancies, specifically, a reasonable proposal would be to consider the whole set of possible arrangements of perinatal conditions with compromised vitality. For instance, 3 groups could be created: both newborn infants with Apgar score < 7; only the first with Apgar score < 7; and only the second with Apgar score < 7. All these analytical approaches may contribute to the resolution of some situations that emerge in the special condition termed twin pregnancy, which remains a challenge for researchers.

## Conclusion

Twin pregnancy is a rare condition that has several particularities and difficulties, not only in the clinical management but also for a scientific approach, making it a challenge for obstetric clinicians and researchers. In comparison to a singleton pregnancy, a twin pregnancy is associated with several maternal complications, including SMM and MNM as well as perinatal mortality and morbidity. The second twin has worse outcomes, possibly due to the delivery, its safety conditions and identification of high-risk groups. This characterizes a major demand for health professionals and centers that are informed and instrumentalized for appropriate prenatal, childbirth and newborn care. On the other hand, the lack of strong definitive and concrete evidence of determinants, associated factors, and consequent maternal and perinatal outcomes of twin pregnancy, in Brazil and across the world, indicate that further studies are needed to specifically address these aims. The major focus should be on population-based studies, with the use of electronic birth registries or large international multicenter studies.
